# Understanding single enzyme activity *via* the nano-impact technique†
†Electronic supplementary information (ESI) available. See DOI: 10.1039/c7sc02084h



**DOI:** 10.1039/c7sc02084h

**Published:** 2017-07-19

**Authors:** Chuhong Lin, Enno Kätelhön, Lior Sepunaru, Richard G. Compton

**Affiliations:** a Department of Chemistry , Physical and Theoretical Chemistry Laboratory , Oxford University , South Parks Road , Oxford OX1 3QZ , UK . Email: richard.compton@chem.ox.ac.uk ; Fax: +44 (0)1865 275410 ; Tel: +44 (0)1865 275957

## Abstract

To evaluate the possible detection of single enzyme activity *via* electrochemical methods, a combined finite difference and random walk simulation is used to model individual enzyme-electrode collisions where such events are monitored amperometrically *via* the measurement of products formed by the enzyme in solution. It is found that the observed signal is highly sensitive to both the enzyme turnover number, the size of the electrode and the bandwidth of the electronics. Taking single catalase impacts as an example, simulation results are compared with experimental data. Our work shows the requirement for the detection of electrochemically active product formed by individual enzymes and gives guidance for the design of experiments.

## Abstract 2

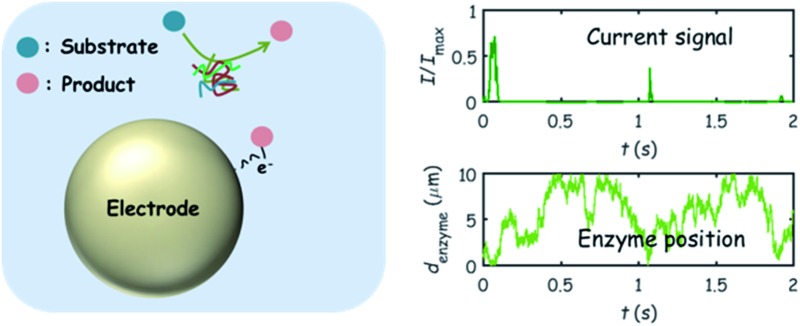
The electrochemical detection and characterisation of individual enzymes *via* the nano-impact technique is predicted.

## 1 Introduction

The nano-impact technique investigates stochastic current signals (“spikes”) that reflect the approach of individual nanoparticles to an electrode and has evolved to become a powerful tool in the analysis of the physical properties as well as the catalytic activity of individual nano-scale particles or macromolecules.^1–6^ In the latter case, a catalyst particle collides with the electrode or is located at or close to the electrode surface and a reaction involving electron transfer is detected, from which the catalytic ability of the particle can be inferred.^7–11^ In the study of enzyme catalysis, the nano-impact technique in principle might enable the observation of enzyme activity at the single-molecule scale while the target enzyme is investigated in its natural environment preserving its original activity and reactivity during the detection.^12–16^ In this respect, the electrochemical method potentially holds an advantage over the conventional spectroscopic methods^17,18^ for studying single enzyme activity, since no enzyme modification is needed. The latter methods, can resolve single catalytic turnover using a single photon counting apparatus. In the ‘nano-impacts’ method the current is the observable. Although electrochemical single electron counting is currently far from realization, information on the flux of charge at variable time scales (in the range μs–s) can be gained, limited by the noise level of the system and the time resolution.

**Fig. 1:**
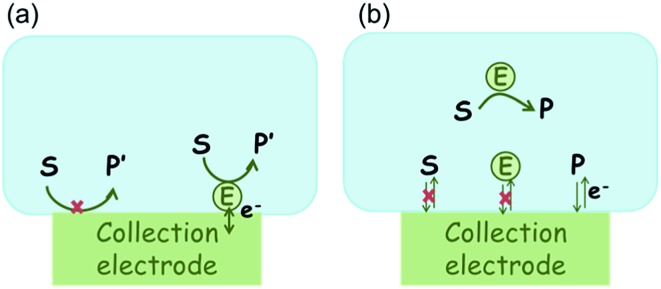
Illustration of two possible enzyme detection methods *via* the nano-impact technique. (a) The enzyme collides with the electrode with which it undergoes direct electron transfer. (b) The catalytic reaction of the enzyme occurs in solution and the product is detected electrochemically.

In the investigation of the activity of an enzyme *via* the nano-impact technique, the detection approach can be classified into two categories: as illustrated in Fig. 1, on the one hand the enzyme activity is measured *via* electron transfer when the enzyme collides with the electrode (Fig. 1a) and on the other it is detected *via* the electrochemical reaction of redox species generated by the enzyme's catalytic reaction in the solution (Fig. 1b). In the first case of an enzyme collision, the catalytic reaction is mediated probably *via* the active site of the enzyme, and the enzyme effectively works as a “nano-electrode” attached to the supporting substrate.^14,19^ The mechanism of the whole process then follows:1
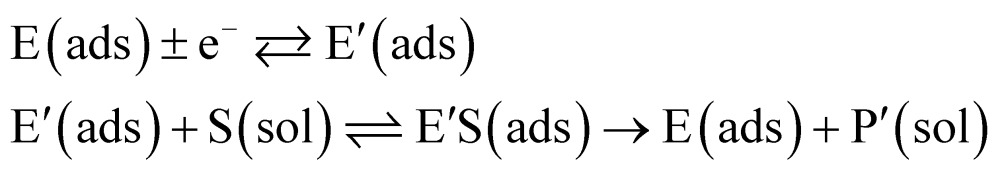
or alternatively:2
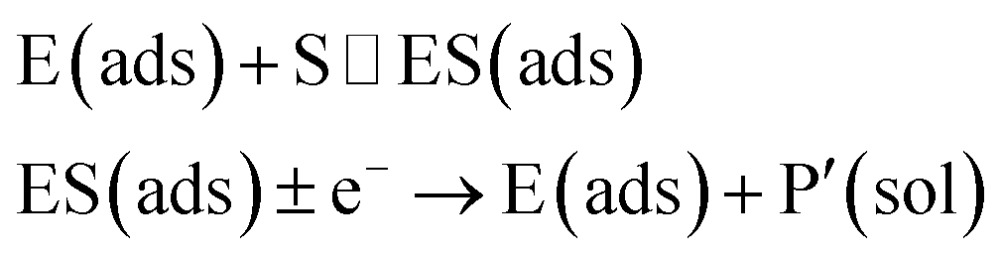
where E and E′ are the original and the reduced/oxidised forms of the active site, and S and P′ are the substrate and the product of the heterogeneous catalytic reaction, respectively. In the second case, the current signal is caused by the electrochemical reaction of the redox species generated by the enzyme. The enzyme is assumed to not necessarily interact adsorptively with the electrode surface but to be solely detected *via* products formed by its reaction with the substrate.^16^ Therefore, the overall process including the catalysis in solution and the detection at the electrode can, assuming Michaelis–Menten kinetics,^20^ be described as:3
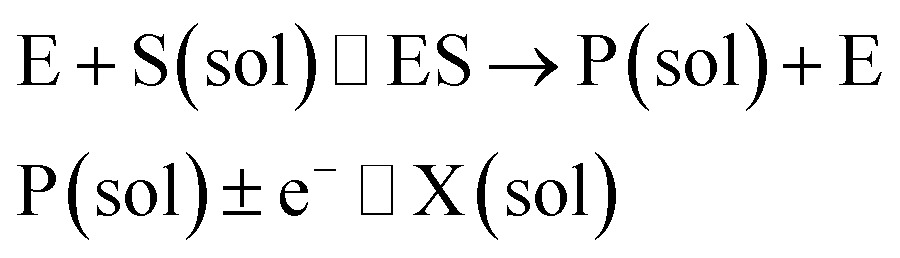
where E is the active site transforming the substrate S into the product P in the solution phase. P is the redox species that reacts at the electrode and X is the reduced/oxidised form of P.

Although current signals corresponding to enzyme activities were observed *via* both methods,^14,16^ the second is probably more suitable for exploring the activity of authentic solution-phase enzyme catalysis as the enzyme does not interact with the electrode surface and any influence of the electrode potential on its active sites can be avoided. Moreover, the product P′ formed by the direct electron transfer to the active site can at least in principle be quite different to that of the solution-phase catalysis P. As the enzyme catalysis was reported to significantly rely on the dynamics of the enzyme and the surrounding reaction environment,^21,22^ it is important to understand the kinetics of the process as otherwise the analysis of corresponding stochastic signals recorded from the electrode remains obstructed and results concluded can at best be exclusively of a qualitative nature.

In this work, a two-dimensional simulation is developed to describe the solution-phase catalysis of the single enzyme. Stochastic current signals (“spikes”) of the detection of the activity of a single diffusing enzyme are simulated. Through comparison of different enzyme-electrode systems, the key factors influencing the measured signal are explored and it is determined under which experimental conditions such experiments may succeed.

## 2 Theory and simulation

The enzyme and electrode are simulated to understand the characteristics of the electrochemical detection of single enzyme activity. To this end and following a short general discussion of enzyme activity (2.1), enzyme catalysis is investigated for a stationary enzyme *via* the finite difference method (2.2), of which the results are then combined with a random walk model for the simulation of the enzyme movement in the solution (2.3) to simulate the electrode response (2.4).

### 2.1 Theoretical model of the enzyme catalysis

The reactions involved in the detection of the solution-phase catalytic reaction of a single enzyme are expressed in eqn (3), where the catalytic activity is examined *via* the reduction or oxidation of the product at the electrode. To simplify the problem, the amount of the substrate S in solution is herein always assumed to be present in excess. The product P is generated from S and the reaction is assumed to follow the Michaelis–Menten kinetics as shown in eqn (5).^20^ The amount of product generated per single enzyme *n*_P_ (mol) is determined by the turnover number *k*_cat_ of the enzyme (s^–1^), the Michaelis–Menten constant *K*_M_ (M), the concentration of the substrate 
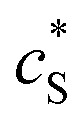
 (M), and the reaction time *t* (s). It is noted that Michaelis–Menten kinetics relate to enzyme ensembles dispersed in the solution and expressed as:4
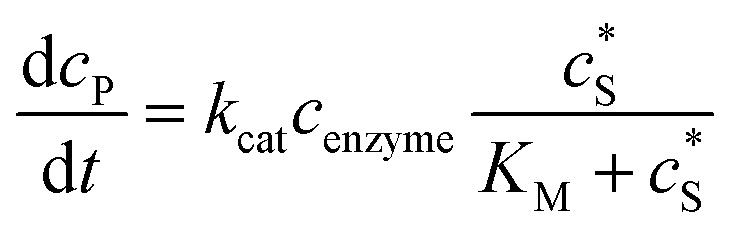
where *c*_enzyme_ is the concentration of the enzyme. We further note that as a starting point, the effect of fluctuations in enzyme activity is not taken into consideration in this model. In the absence of dynamic disorder, the average activity over time and the average activity of an ensemble of enzymes are hence equivalent.^23^ Application of eqn (4) to the catalysis of a single enzyme then yields:5
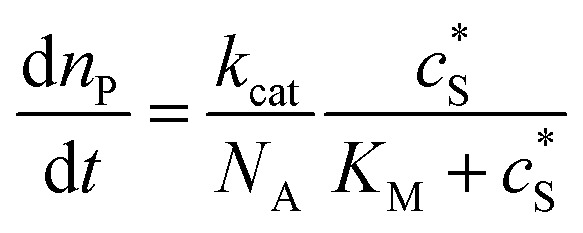
where *N*_A_ is the Avogadro constant (6.022 × 10^23^ mol^–1^). Through the application of Fick's first law, it is found that the concentration of P *c*_P_ (mM) is determined by the catalytic ability of the enzyme and the mass transport of P:6
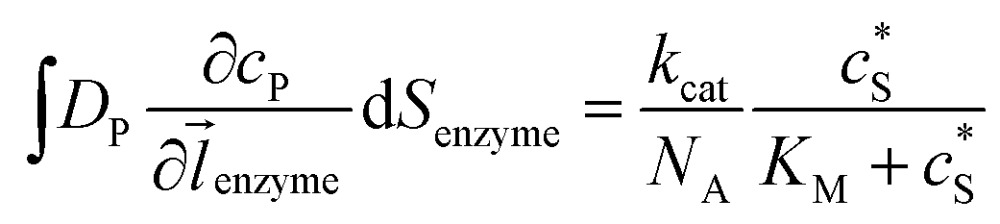
where *D*_P_ is the diffusion coefficient (m^2^ s^–1^) of P, *l*_enzyme_ is the unit vector pointing from the enzyme surface, 
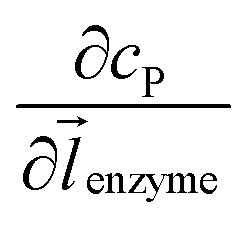
 is the concentration gradient (M m^–1^) of P on and perpendicular to the enzyme surface, and *S*_enzyme_ is the surface area of the enzyme (m^2^).

If the reaction environment contains enough supporting electrolyte and the detection time is relatively short, only diffusion needs to be considered when modelling the mass transport.^24,25^ Here we assume that a single enzyme generates enough product so that the distribution of product molecules can be properly described as a concentration rather than individual molecule positions. The diffusion of the product is then described *via* Fick's second law:^26^
7
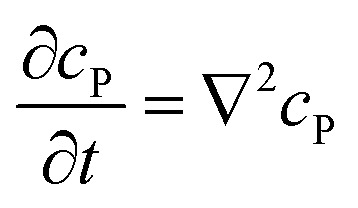



Assuming the over- or under-potential applied at the collection electrode is high enough to immediately consume all product species reaching the electrode surface, the concentration of P at the electrode surface is regarded to be effectively zero during the experiment. The current arising from the reduction or oxidation of P can then be calculated from the concentration gradient at the electrode surface:8
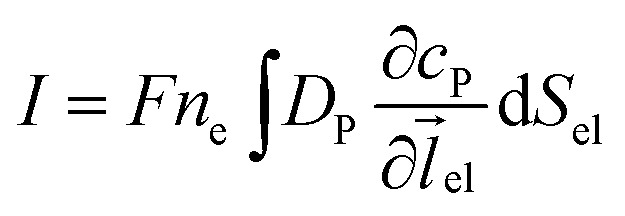
where *F* is the Faraday constant (96 485 F mol^–1^), *n*_e_ is the number of electrons transferred per product (in this work, *n*_e_ is set to be 1), *l*_el_ is the unit vector pointing from the electrode surface, and *S*_el_ is the surface area of the electrode.

### 2.2 Simulation of the electrode response to a stationary enzyme

**Fig. 2:**
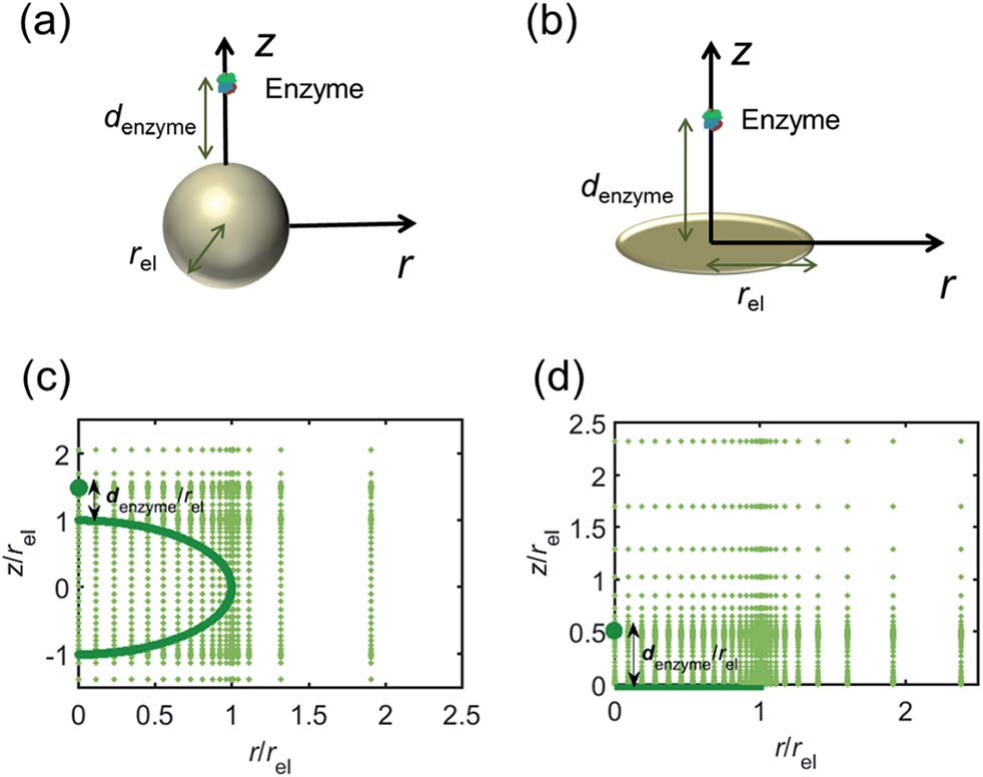
Simulation model for the detection of single enzymes. (a) Illustration of a single enzyme near a sphere electrode; (b) illustration of a single enzyme near a disc electrode; (c) simulation mesh for the model in (a); (d) simulation mesh the model in (b).

For simulating the catalysis of a stationary enzyme, the finite difference approach is applied. As the size of the enzyme is small compared to the size of the detecting electrode, the enzyme is treated as a point in the simulation space. To simplify the problem, the orientation of the enzyme is not taken into consideration and a micro-size spherical electrode is first selected as the detecting electrode. The simulation space can then be described in two-dimensional cylindrical coordinates (*r*, *z*). As shown in Fig. 2a, the *z* axis is located on the line linking the enzyme and the centre of the spherical electrode, and the *r* axis is set perpendicular to the *z* axis. In addition and to further simplify the enzyme-electrode system as explained below, in a second simulation the spherical electrode is replaced by a disc electrode in the same coordinate system, as illustrated in Fig. 2b.

For the numerical simulation *via* the finite difference method, meshes of both the sphere- and the disc model are built, of which two examples are shown in the Fig. 2c and d and on which more details can be found in the ESI.† The conditions and equations used to describe the enzyme-electrode system are listed in Table 1.

**Table 1:** List of the initial condition, boundary conditions and the partial differential equations for the enzyme-electrode model

Condition	Equation
*t* = 0	*c*_P_ = 0
*r* → ∞	*c*_P_ = 0
*z* → +∞/–∞	*c*_P_ = 0
Spherical electrode: *z*^2^ + *r*^2^ = *r*_el_^2^	*c*_P_ = 0
Disc electrode: *r* ≤ *r*_el_, *z* = 0	
Spherical electrode: *r* = 0, |*z*/*r*_el_| > 1	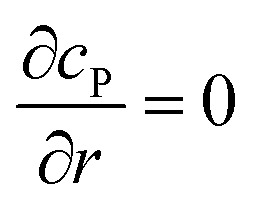
Disc electrode: *r* = 0	
Disc electrode: *z* = 0, *r*/*r*_el_ > 1	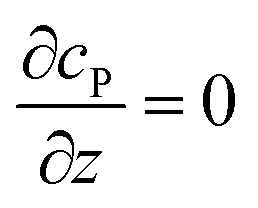
Spherical electrode: *r* = 0, *z* = *r*_el_ + *d*_enzyme_	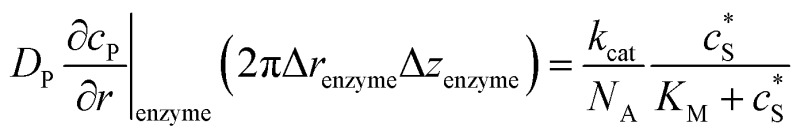
Disc electrode: *r* = 0, *z* = *d*_enzyme_	
*r*, *z* in the solution	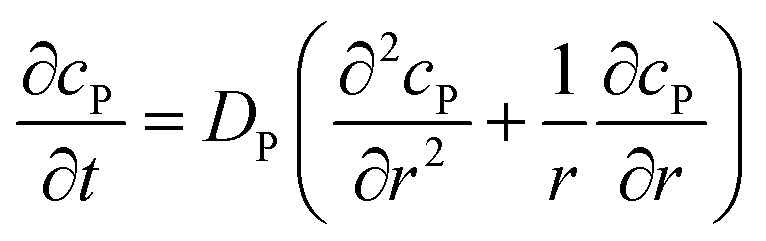

To numerically simulate the enzyme catalysis based on the theoretical model described by eqn (6), the enzyme is treated as a point and the process can be described as:9
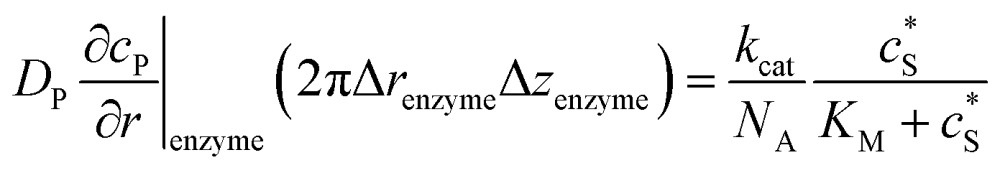
where Δ*r*_enzyme_ and Δ*z*_enzyme_ are the space intervals between the enzyme point and the adjacent grid points in *r* and *z* direction, respectively. We note that as the enzyme is assumed to be a point source, the homogeneous product flux 
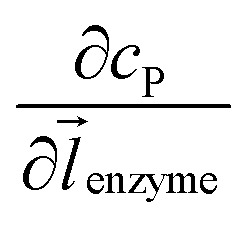
 can be approximated to be 
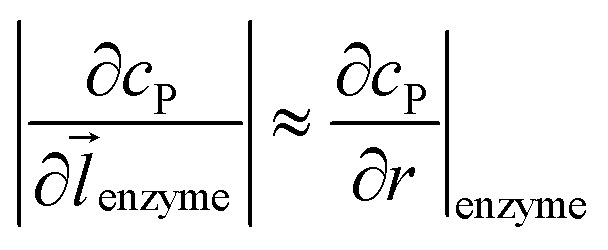
 and only one direction is evaluated as shown in eqn (9).

Through the finite difference method, the enzyme catalysis can be expressed as:10
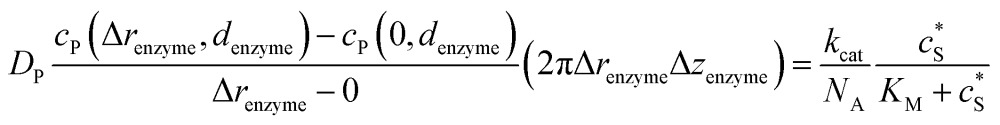



With the above definition the simulation is convergent as the choice of Δ*r*_enzyme_ and Δ*z*_enzyme_ does not influence the results (the corresponding convergence tests can be found in the ESI†).

In this work, we define three dimensionless properties to characterise the single-enzyme catalysis: the flux at the electrode *J*, the total amount of product generated by the enzyme *N*_P_, and the “collection efficiency” of the electrode *σ*:11
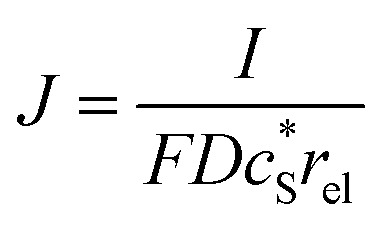

12
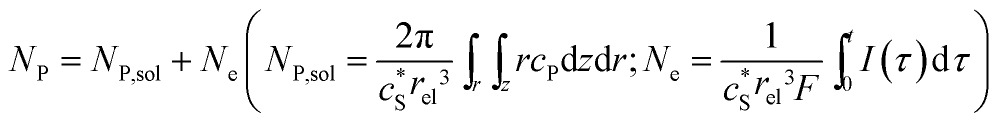

13
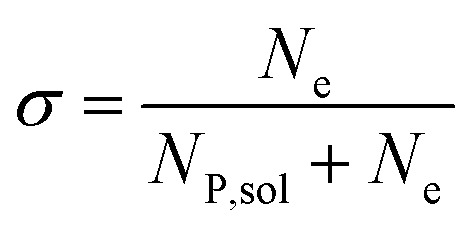
where *N*_P,sol_ is the amount of the product in the solution and *N*_e_ is the amount of product consumed at the electrode until the time *t*.

### 2.3 Simulation of the enzyme diffusion

In the above finite difference model, which focusses on the diffusion of the product, the enzyme is treated as a stationary point at a fixed distance *d*_enzyme_ from the electrode. However, due to its Brownian motion, the enzyme does not remain at a fixed position but randomly moves in solution. The enzyme is therefore treated as a random walker when modelling its movement in the electrolyte. When the it enters the region close to the electrode, the enzyme can be detected *via* its catalytic product, which partly diffuses towards to the electrode where it may be oxidised or reduced, and a corresponding current “spike” may be observed in the chronoamperogram.^15,16^ The diffusion of the enzyme is herein dependent on the distance between the enzyme and the electrode surface, which is due to the effect of near-wall hindered diffusion.^27–30^

If we only consider the diffusion of the enzyme perpendicular to the electrode surface, the random walk of the enzyme can be simulated in one direction *x*, defined as the dimension perpendicular to the electrode surface. In the hindered diffusion theory, the distance-dependent diffusion coefficient of the enzyme can be expressed as:^31^
14
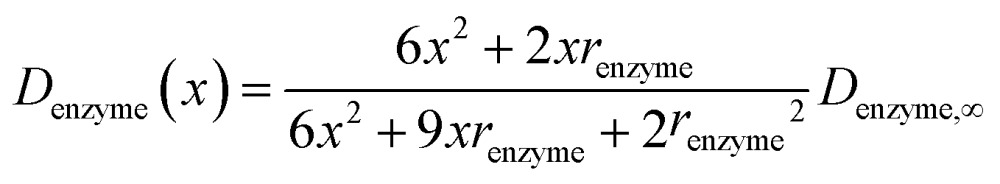
where *r*_enzyme_ is the radius of the enzyme and *D*_enzyme,∞_ is its diffusion coefficient in bulk solution. Here it needs to be noted that when focusing on the movement of a single enzyme, the enzyme is no longer regarded as a point but treated as a nano-sphere with certain volume, where the radius of the enzyme can be approximated from the volume of the enzyme. We further note that the above equation only applies to the diffusion towards a plane and is here used as an approximation.

The diffusion of the enzyme follows Fick's second law:15
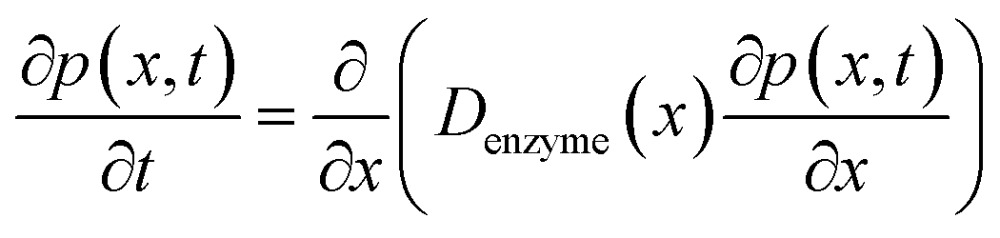
where *p*(*x*, *t*) is the probability distribution of the enzyme position. The probability distribution at the beginning of each random walk step is described by a Dirac delta function:

16 *p*(*x*) = *δ*(*x* – *x*_0_)

where *x*_0_ is the location of the enzyme. By solving eqn (15), the probability distribution after one random walk step *p*(*x*, Δ*t*_rw_) (Δ*t*_rw_ is the time interval between two random movements) can be determined. The average displacement Δ*x*_0_ is then calculated as:17
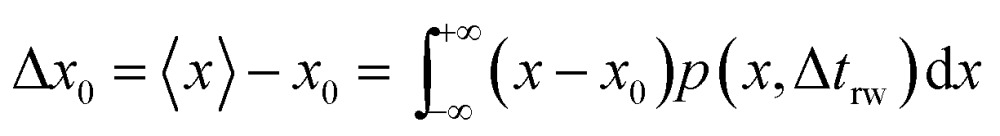
and the average absolute displacement Δ*x*_±_ is calculated from the standard deviation:18
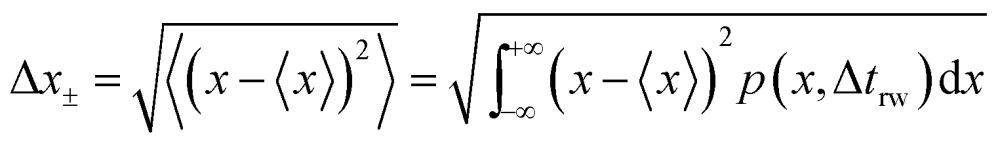



The direction of the movement after each random walk is herein set randomly and the total displacement hence:19
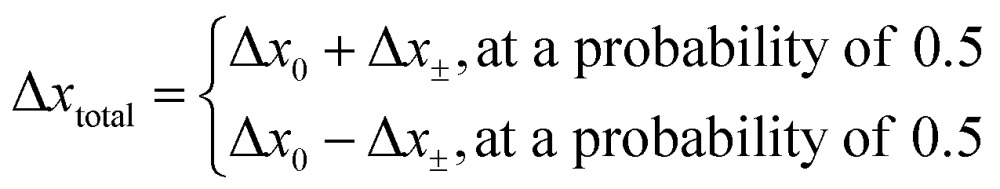



The location of the enzyme after the *k*th random walk step is then determined as:

20 *x*_0_^*k*^ = *x*_0_^*k*–1^ + Δ*x*_total_

However, although the random walk step length Δ*x*_total_ can be calculated *via* solving the mass transport equation eqn (15), the simulation is very time-consuming, especially when the level of accuracy required is high and the modelled time interval is long. Therefore, in order to optimise the simulation procedure, an approximation to the random walk is used.

If the diffusion is homogeneous, that is *D*(*x*) = *D*_enzyme,∞_, the probability distribution after each random walk step is a Gaussian function and the step length of each random step is 
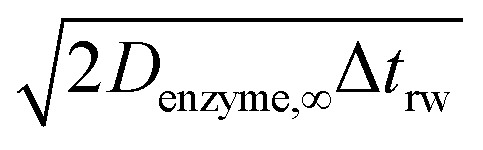
.Taking hindered diffusion into account, the Gaussian step length for the one-dimensional random walk however needs to be corrected. It is reported in the literature that the corrected Gaussian step length Δ*x*_total_ for the case of anisotropic diffusion can be expressed as:^32,33^
21
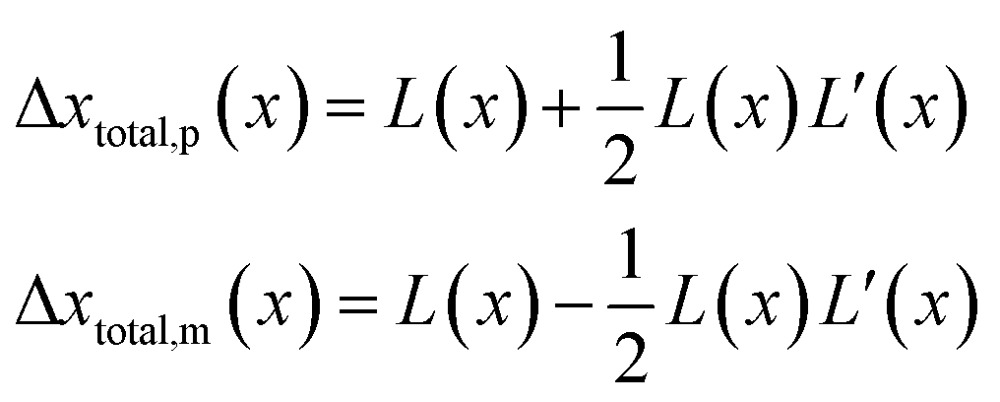
with the corresponding probabilities22
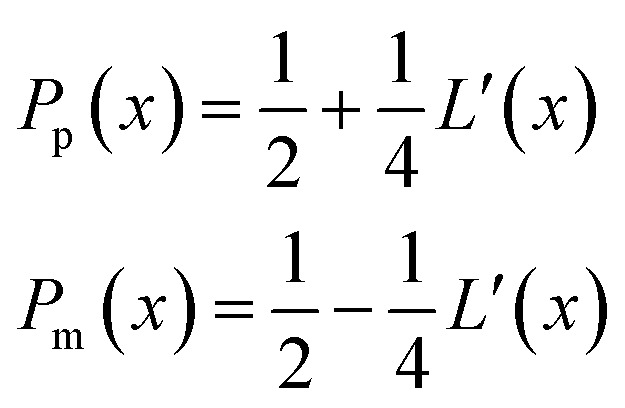
where the subscripts p and m refer to the two directions of the one-dimensional random walk. *L*(*x*) = 
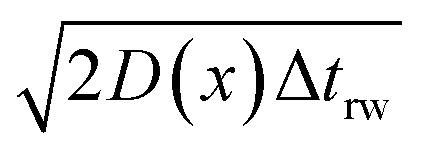
 is the uncorrected step length and *L*′(*x*) = d*L*(*x*)/d*x*.

### 2.4 Simulation of the electrode response to a diffusing enzyme

For a time series of enzyme locations {*d*_enzyme_^*i*^} = {*d*_min_, … *d*_max_}, the corresponding catalytic currents {*i*_enyzme_^*i*^(*d*_enzyme_^*i*^, *t*)} can be calculated from the enzyme catalysis model introduced in Section 2.1, where the discretised *i*_enzyme_ signify the average current between two sampling points. For each *i*_enyzme_^*i*^(*d*_enzyme_^*i*^, *t*), which is the current arising from an enzyme located at a fixed position and being active within the time interval [0, Δ*t*_rw_], the catalytic reaction is modelled, while significantly longer time interval are considered as the electrode response to the product generated within this time interval is at least partly observed after *t* = Δ*t*_rw_. On the basis of these {*i*_enyzme_^*i*^(*d*_enzyme_^*i*^, *t*), *d*_enzyme_ ∈ [*d*_min_, *d*_max_]}, the current contribution of each random walk step can be approximated *via*:23
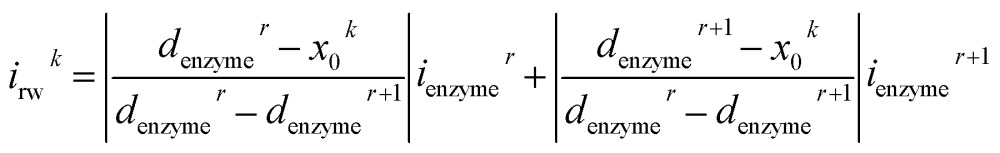
where *i*_rw_^*k*^ is the current during the *k*th random walk step, *x*_0_^*k*^ is the enzyme location determined after the *k*th random walk step. *d*_enzyme_^*r*^ and *d*_enzyme_^*r*+1^ are the pre-defined enzyme position adjacent to the simulated enzyme location *x*_0_^*k*^, *d*_enzyme_^*r*^ ≤ *x*_0_^*k*^ < *d*_enzyme_^*r*+1^. *i*_enzyme_^*r*^ and *i*_enzyme_^*r*+1^ correspond to the enzyme positions *d*_enzyme_^*r*^ and *d*_enzyme_^*r*+1^. The total current at the electrode that includes the contributions from every former enzyme position can be calculated as:24
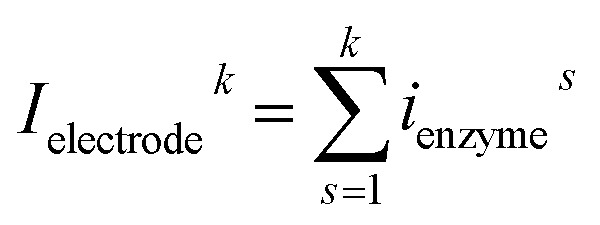



It is herein noted that the recorded spike shape is also determined by the measurement sampling frequency and the filter built into the potentiostat.^34^ The filter is of particular relevance as it may lower the height of the observed peak currents if the electrode signal exceeds the filter bandwidth. Measured currents hence represent a lower limit for the actual electrode currents if the measured spike shape is similar to the filter's impulse response, which for instance is the case in our previous work.^16^ To model a more realistic experimental condition, the current calculated from the random walk of the enzyme is therefore filtered *via* a first-order Butterworth filter.^35^

The finite difference problem is solved numerically by means of the Newton–Raphson method and the alternating direction implicit (ADI) method.^36^ The simulation is written in Matlab R2016a and run on an Intel(R) Xeon(R) 3.60G CPU. The validation of the simulation program is examined *via* the convergence tests which can be found in the ESI.† ^37^

## 3 Results and discussion

This section studies the catalysis of a single enzyme near a micro-electrode and explores the possibility of its detection *via* the electrochemical reaction of the product molecules. We herein first investigate the electrode response to a stationary enzyme (3.1) before we model amperometric currents resulting from a freely-diffusing enzyme (3.2). The latter is accompanied by experimental data of catalase and signals originating from both experiments and simulations are compared (3.3). Finally, we provide a discussion of the implications of our findings on the design of experimental set-ups.

### 3.1 Electrode response to a stationary enzyme

Fig. 2 illustrates the two microelectrode geometries that are considered in the simulation of the enzyme-electrode system. Numerical results reveal that, although absolute values of the currents collected from the two microelectrodes are not identical; their responses are similar as illustrated in the ESI.† The computationally more efficient enzyme-microdisc system can therefore be treated as an excellent approximation for the enzyme-microsphere electrode and, in the following, all simulation results are based on the enzyme-microdisc model, while similar conclusions can be drawn with regard to the enzyme-microsphere system.

We characterise the enzyme-electrode system by the flux *J* (defined in eqn (11)), the total amount of product generated by the enzyme *N*_P_ (eqn (12)), and the collection efficiency of the electrode *σ* (eqn (13)). To illustrate the catalysis-collection process, three dimensionless quantities affecting the current collected by the electrode are further introduced: the enzyme catalytic ability, *K*_cat_, the relative distance between the enzyme and the centre of the electrode *d*, and the normalized reaction time *T*. The dimensionless parameter *K*_cat_ is defined as:25
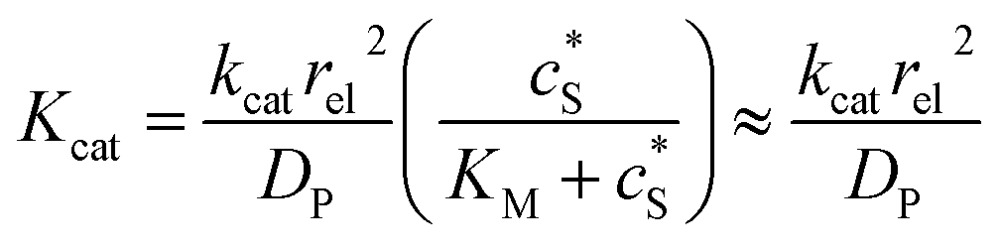



In this paper, where it is assumed that the enzyme is always exposed to an excess concentration of the substrate, 
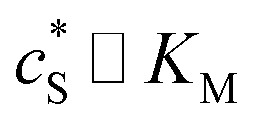
 and thus 
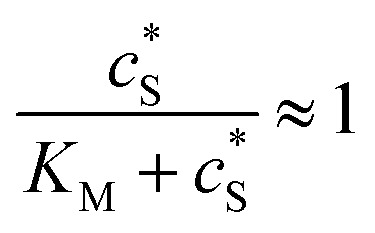
. The relative distance between the enzyme and the centre of the microdisc electrode is normalized with respect to the size of the electrode:26
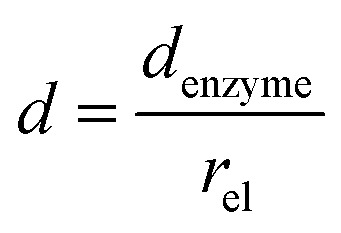
where *d*_enzyme_ (m) is the absolute distance from the centre of the electrode to the enzyme location. *d* reflects the mass transport of the enzyme product from the enzyme to the electrode. *T* refers to the reaction time, normalized to the radius of the electrode *r*_el_ and the diffusion coefficient of the product *D*_P_:27
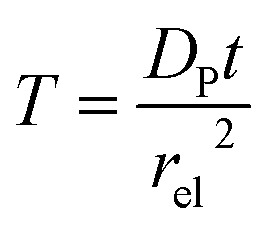



The advantage of using these “combined” parameters is that all variables, such as *r*_el_, 
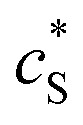
 and *D*_P_, are grouped according to their influence on the enzyme-electrode system. It is then clearer to describe the characteristics of the whole process.

**Fig. 3:**
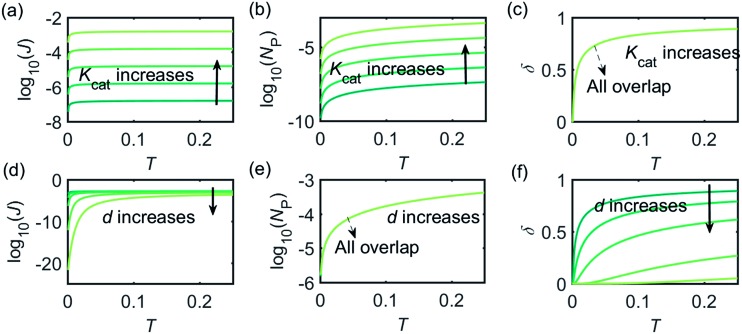
Characterisation of the enzyme-microdisc system. (a–c) are the current flux *J*, the total amount of product *N*_P_, and the collecting efficiency *σ* varying with the reaction time *T* at different catalytic abilities *K*_cat_ from 10 to 10^5^. *d* = 0.05. (d–f) Depict the current flux *J*, the total amount of product *N*_P_, and the collecting efficiency *σ* varying with the reaction time *T* at different enzyme locations *d*. *d* varies from 0.05 to 1.0. *K*_cat_ = 10^5^.

Fig. 3 illustrates how the enzyme-electrode system is affected by these factors. Fig. 3a–c show the flux *J*, the amount of product *N*_P_, and the collection efficiency *σ* varying as a function of *T* at different enzyme catalytic abilities *K*_cat_. It can be seen that *J* and *N*_P_ are determined by *K*_cat_ while *σ* is independent on the value of *K*_cat_. In Fig. 3d–f, the influence of the relative enzyme location *d* is examined. *J* and *σ* are found to be affected by the value of *d* while *N*_P_ remains constant when the enzyme changes its location. When the reaction time is long enough, the enzyme-electrode system is able to reach steady state, where the flux no longer increases with time. The total amount of the enzyme product *N*_P_ at the steady state is determined only by the catalytic ability *N*_P_ = *f*(*K*_cat_), the collection efficiency is only related to the enzyme location *σ* = *f*(*d*), and the reaction flux is a function of both factors *J* = *f*(*K*_cat_, *d*).

**Fig. 4:**
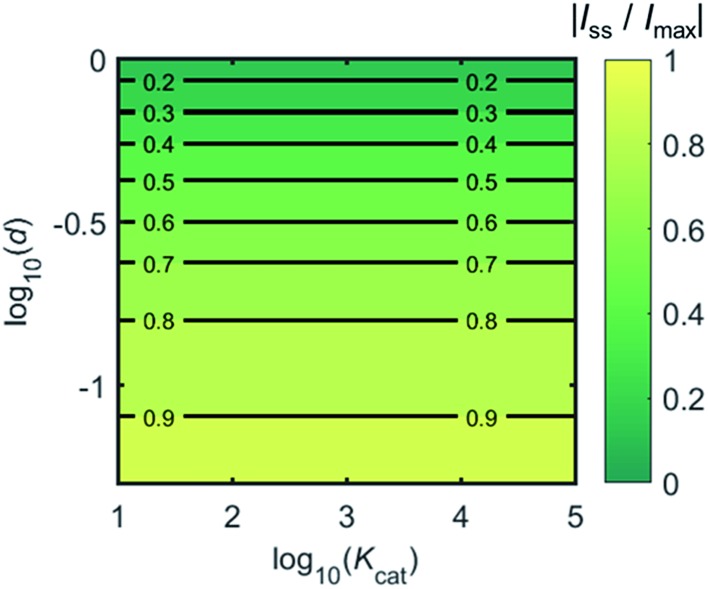
Normalized steady-state current (defined as the current at *T* = 0.25) as a function of *d* and *K*_cat_ for the enzyme-microdisc system.

Fig. 4 shows a working curve of the normalized current at the steady state varying as a function of *d* and *K*_cat_. For clarity, the steady-state current *I*_ss_, which is defined as the current value at *T* = 0.25, is normalized by *I*_max_. *I*_max_ is the maximum current that can be collected by the electrode and is limited by enzyme catalysis, corresponding to the case where the enzyme locates exactly at the electrode surface and each product molecule generated is immediately consumed by the electrode. Thus *I*_max_ can be predicted by the turnover number of the enzyme:

28 *I*_max_ = *k*_cat_*e*_0_

where *e*_0_ is the charge on an electron, 1.602 × 10^–19^ C. It can be seen from Fig. 4 that after the normalization, *I*_ss_/*I*_max_ is independent from the catalytic kinetics but only influenced by the distance from the electrode, indicating that compared to the fast catalysis of the enzyme, the diffusion of the product is the rate-limiting process in the enzyme-electrode system.

### 3.2 Electrode response to a freely-diffusing enzyme

When the enzyme movement is additionally taken into consideration, the generated product does not fully reach the electrode before the enzyme moves on to its next position and both processes, the product diffusion and the enzyme movement, are modelled separately. The enzyme is herein treated as a random walker, while the step length Δ*t*_rw_ of the random walk is determined by convergence tests to ensure that Δ*t*_rw_ is small enough in comparison with the bandwidth of the simulated measurement electronics (1/*f*_cutoff_). Please note that below dimensional variables (*i.e. r*_el_, *D*_P_, *d*_enzyme_) are depicted in the modelling of real nano-impact experiments, while the above Fig. 3 and 4 employ dimensionless parameters (*i.e. J*, *K*_cat_, *d*) to better illustrate the kinetics of the stationary enzyme.

**Fig. 5:**
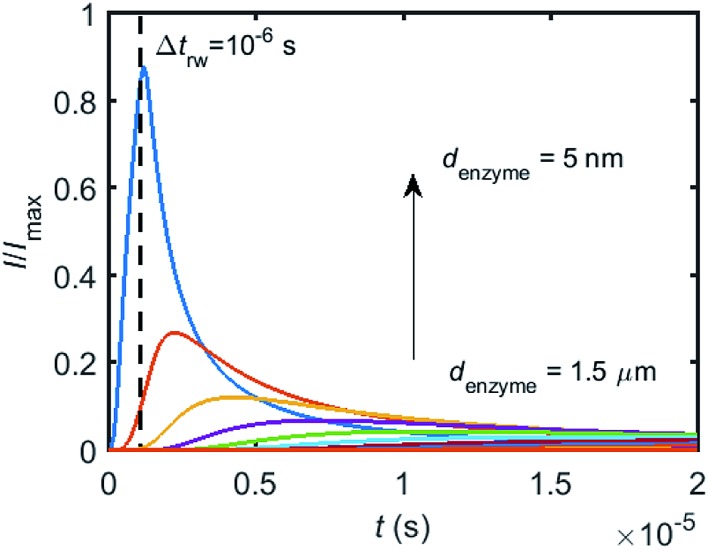
Collection of the normalised current–time response to a single enzyme at a series of enzyme positions. The dashed line shows the random walk step length Δ*t*_rw_ = 10^–6^ s. The enzyme positions *d*_enzyme_ shown in the figure range from 5 nm to 1.5 μm. *r*_el_ = 0.5 μm, *D*_P_ = 10^–9^ m^2^ s^–1^.

To simulate the chronoamperogram for a diffusing enzyme, the electrode's current responses to stationary enzymes are collected for a series of enzyme locations as discussed in Section 2.4 and exemplarily shown Fig. 5 where the current responses to an enzyme at locations *d*_enzyme_ ranging from 0.05 to 1.5 μm are modelled. The enzyme herein features a size *r*_enzyme_ of 5 nm and a bulk diffusion coefficient *D*_enzyme,∞_ of 5 × 10^–11^ m^2^ s^–1^, while the microdisc radius is set to 0.5 μm and the diffusion coefficient of the product *D*_P_ is set to 10^–9^ m^2^ s^–1^. To generalise the results, the collected current is normalized to the maximum catalytic current *I*_max_. For each current response in Fig. 5, the catalytic reaction only occurs within the time period 0 < *t* ≤ Δ*t*_rw_ in which the enzyme is stationary. The current at *t* > Δ*t*_rw_ is caused by the fraction of the product which is not fully consumed during the time 0 < *t* ≤ Δ*t*_rw_. It can be found in Fig. 5 that the closer the enzyme is located to the electrode, the larger is the catalytic current and the sharper is the drop of the current amplitude after Δ*t*_rw_ is reached. For all further simulations the stationary current response was modelled in a time interval *t*_max_ of 10^–4^ s *via* the finite difference simulation, while the response in the range *t*_max_ < *t* < 2*t*_max_ is interpolated linearly to *i*_enzyme_ (2*t*_max_) = 0. The convergence tests of *t*_max_ can be found in the ESI.†

**Fig. 6:**
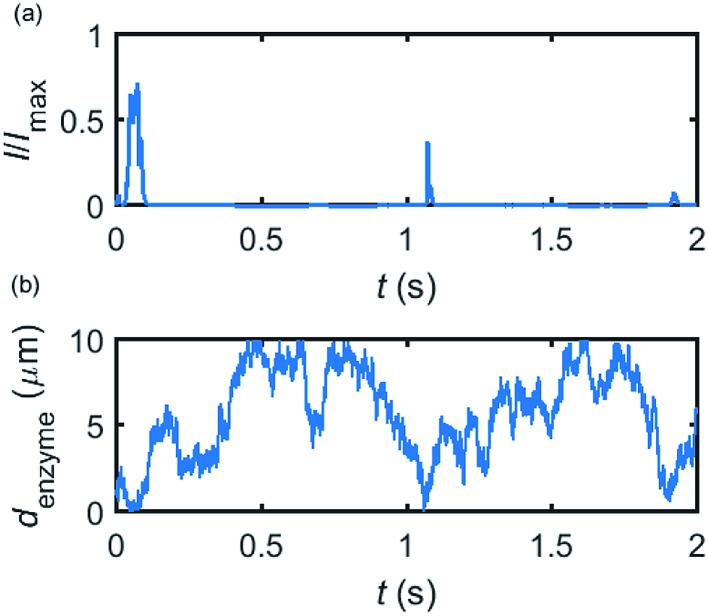
Example of a simulated random walk current (a) and the corresponding pathway in the solution (b). The space, in which the enzyme moves freely, ranges from 5 nm to 10 μm. Other simulation parameters are the same as applied in Fig. 5.

Based on the normalized current–time responses shown in Fig. 5, the chronoamperogram of the diffusing enzyme is simulated in Fig. 6 and the corresponding pathway of its movement is presented. Two current “spikes” can be observed, while each spike indicates an approach of the enzyme towards the electrode and the spike shape infers the details of each approach. When the enzyme immediately leaves the electrode after an approach, a sharp spike is measured in the chronoamperogram such as that at around 1.1 s in Fig. 6. On the other hand, if the enzyme moves forward and backward several times near the electrode, a long spike with noisy current fluctuations will be recorded, such as the one at 0–0.1 s.

Fig. 6 proves that the solution-phase enzyme catalysis can in principle be observed experimentally. It is also shown that each approach of the enzyme can be distinguished as the current is very sensitive to changes in the distance between the enzyme and the electrode, which enables a further analysis of the spike data and the extraction of information on both the enzyme catalysis and the enzyme diffusion. This two dimensional result differs significantly from the one-dimension case discussed in the previous work:^15^ in the two-dimensional case, convergent diffusion leads to a collection efficiency that depends sharply on the distance between the enzyme and the electrode. When the enzyme diffuses towards or away from the microelectrode, sharp current on- and offsets can be observed in the chronoamperometry that are due to the dependency of the collection efficiency on the enzyme location as shown in Fig. 3 and 4. This sensitivity of the microelectrode in principle enables the detection of single enzyme activity *via* its product in the nano-impact technique, which is obscured in the case of a macroelectrode. The semi-infinite diffusion field at the macroelectrode resulting from linear diffusion and coupled to the marked mismatch of the diffusion coefficients of the enzyme and its product means that the 'collection' of the product is much less sensitive to the motion of the enzyme.

**Fig. 7:**
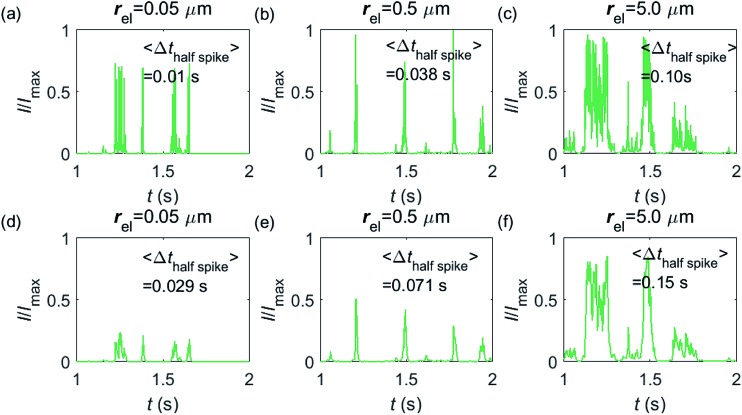
Chronoamperograms of single enzyme detection at microdisc electrodes featuring radii of 0.05, 0.5 and 5.0 μm. (a–c) are the modelled potentiostat signals after passing a 4 kHz filter, while (d–f) depict the same data filtered *via* a 50 Hz filter. The average spike duration time for each system is listed on the plot. In the simulation, *r*_enzyme_ = 5 nm, *D*_enzyme_ = 5 × 10^–11^ m^2^ s^–1^, *D*_P_ = 1 × 10^–9^ m^2^ s^–1^, Δ*t*_rw_ = 10^–6^ s. The simulation space of *d*_enzyme_ is from 5 nm to 5 μm and the total simulation time is 5 seconds.

Having shown that spikes can only be observed at micro-sized electrodes, the influence of the electrode size needs to be taken into consideration. Fig. 7 shows the current responses to a freely-diffusing enzyme at microdisc electrodes of various radii. Fig. 7a–c are the chronoamperograms (normalized to the maximum possible current, *I*_max_) at electrodes featuring radii of 0.05, 0.5 and 5.0 μm and the cut-off frequency of the measurement filter is set to 4 kHz; (d–f) are the same simulations as shown in (a–c) but with a different cut-off frequency of 50 Hz. The spike durations in each chronoamperogram are analysed and indicated in the corresponding figure. The spikes are recognized *via* a threshold value that is set to distinguish a spike from the background. In the analysis in Fig. 7, the threshold value for spike duration is selected to be 0.5% of the maximum spike current. In addition, considering the noise level in the real experiment, spikes featuring a small current height are unrecognizable and are here removed if the peak current is less than 10% of the maximum spike current. To avoid any influence from the background noise, the spike duration is characterised by the width at half of the spike height Δ*t*_half-spike_.

From Fig. 7a–c, it is found that the spike features a height close to the maximum possible current *I*_max_ at the electrodes with 0.05, 0.5 and 5.0 μm radii. The current signal at the 0.05 μm is slightly smaller than the other two due to the finite closest approach of the enzyme applied in the simulation. The closest approach *d*_min_ is defined as the radius of the enzyme (5 nm) for all the three electrode systems but the magnitude of the current spike is determined by the relative closest approach *d*_min_/*r*_el_ as explained in Fig. 3 and 4. Therefore, the spikes recorded at the 0.05 μm electrode (*d*_min_/*r*_el_ = 0.1) are slightly lower than that at the 5.0 μm one (*d*_min_/*r*_el_ = 0.001). More discussion on the selection of the simulation space can be found in the ESI.† It is also found in (a–c) that the spike length varies significantly with the electrode size. The average half-spike widths of 0.05, 0.5 and 5.0 μm electrodes are 0.01, 0.038 and 0.10 s under the 4 kHz cut-off frequency. Sharper spikes are observed at the 0.05 μm electrode than the 5.0 μm electrode, reflected by a 5 fold decreases in the spike duration. This is because the current is more sensitive to the variation of the enzyme location at the smaller electrode. The spikes last for longer at larger electrodes, showing the transition from the two-dimensional to the one-dimensional system as discussed above.

In Fig. 7d–f, with a 50 Hz cut-off frequency, a similar dependency of the current signal on the electrode size is still observed as shown in (a–c). The spikes recorded at larger electrodes have longer duration time. However, the spike height in (d–f) is smaller than *I*_max_, especially at the smallest 0.05 μm electrode. This is because that a low cut-off frequency is employed and the current response recorded from the electrode is largely distorted. Comparison of the chronoamperograms in (a–c) and (d–f) reveals that the spike currents in (a–c) are larger than those in (d–f) and the spikes are sharper, as a low-pass filter with higher cut-off frequency retains more information of the original current recorded.

### 3.3 On the possible electrochemical detection of single catalase enzymes

Based on the model developed in this work, the electrochemical detection of single catalase enzymes is simulated. To this end, the experimental parameters of the catalase-microdisc system are modelled: *D*_P(O2)_ = 10^–9^ m^2^ s^–1^,^38^ *r*_el_ = 5 μm,^15^ *D*_enzyme(catalase)_ = 5 × 10^–11^ m^2^ s^–1^,^39^ *k*_cat(catalase)_ = 10^6^ s^–1^,^40^ *r*_enzyme(catalase)_ = 5 nm,^41^ *f*_cutoff_ = 4 kHz.^15^ In reported work of this group, single catalase impact experiments were conducted in a 9 pM catalase solution with chronoamperograms recorded at a 5 μm radius microdisc electrode.^15^ The reason for choosing catalase is mainly due to its high turnover number^40^ which is at the upper limit of known enzymatic catalytic rates. In addition, the oxygen product is easy to detect electrically.

**Fig. 8:**
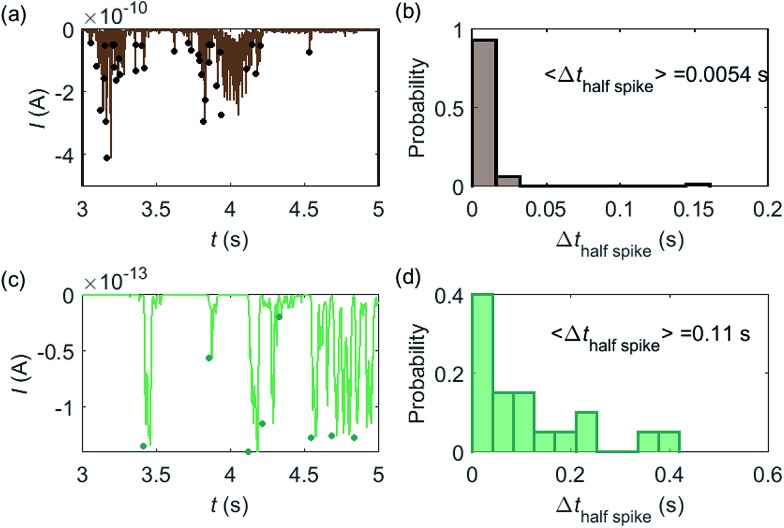
(a) is an experimentally found chronoamperogram of 9 pM catalase in a 100 mM hydrogen peroxide solution at an applied potential of –1.0 V *versus* SCE, measured at a 5 μm radius microdisc electrode; (b) is the corresponding histogram of the half-spike width of the current spikes in (a); (c) and (d) are the simulated chronoamperograms (see text) referring to single catalase detection at a microdisc electrode and the corresponding histogram of the half-spike width. The total recording time is 50 s for both experiment and simulation. The simulation space is from 5 nm to 10 μm.

Some typical current spikes collected from the experiment are shown in Fig. 8a and the analysis of the spikes is presented in Fig. 8b. The control experiment (see Fig. S8 in the ESI†) relating to Fig. 8a in the absence of catalase does not show any spikes in the chronoamperometric measurements, indicating that the spikes relate to the catalase catalysis. From the experimental results, the magnitude of the spike height is approximately 10^–10^ A and the average half-spike width is 0.0054 s. For direct comparison, a simulation of the same catalase-microdisc system is shown in Fig. 8c and d. The simulated spikes feature a height of *ca.* 10^–13^ A and an average half-spike width of 0.11 s. To compare the experimental and the simulated current responses, we consider both the duration and shape of the spike. First, the simulated half-spike width Δ*t*_half-spike_ is significantly longer than that of the experiment. According to the simulation in Fig. 7, only broad spikes are anticipated to be observed at the relatively large 5 μm electrode, which contradicts experiment. Secondly, as the magnitude of the spike height is mainly determined by the turnover number and the reported turnover number (10^6^ s^–1^) measured from an ensemble of catalases^40^ leads to spike heights of the order of 10^–13^ A, the simulated spikes are much too small to be distinguished from the background noise in any real experiment using broad bandwidth. That said, in contrast to the kinetics averaged over an ensemble as reflected in the classical Michaelis–Menten kinetics, single enzyme activity is thought to be dynamically fluctuating and the turnover number for individual enzymes can deviate from the average value.^22,42^ Hypothetically, if the turnover number of a single catalase is temporarily near 10^9^ s^–1^ at the moment of detection, a current spike of 10^–10^ A can be observed, which is experimentally feasible to measure. However, the activity of single catalase is yet to be reported by optical or other experimental means and the fact that the enzyme contains four catalytic heme centres probably slightly averages any dynamic disorder of the enzyme catalytic rate. Comparison of the experimentally measured current (Fig. 8a) and the theoretical calculated current spikes (Fig. 8c) reveals a three orders of magnitude difference in the current magnitude. This discrepancy might be explained by experimental artefacts such the formation of electrochemically active oxygen bubbles.^15^ Alternatively, the experimental spikes may reflect enzymatic activity operating ‘transiently’ at catalytic rates that are three orders of magnitude higher than the ensemble averaged rate and consequently with substantially lower observed “impact” frequency. However, this process can be further complicated by contribution from surface adsorbed enzymes and is out of the scope of this theoretical investigation. In either case, full information on the enzyme turn-over rate cannot be gained.

### 3.4 Implications for the design of experiments

The possibility of experimentally detecting the activity of an individual enzyme in solution is mainly determined by the maximum current and the duration time of the signal. If under experimental conditions a 10 pA current spike is the minimum current that can still be observed at a microelectrode, according to eqn (28), the turnover number needs to be of the order of magnitude of 10^8^ s^–1^ in the case of one electron being transferred per consumed substrate molecules, which means the detection of the activity for an individual enzyme is feasible for enzymes exhibiting a fast turnover number or an agglomerate or aggregate of enzymes.^16^ In addition, the spike duration determines whether a signal can be distinguished from the background noise. Only sharp spike onsets can be identified, while slow spike on- and off-sets will be indistinguishable from (the typically slow) changes in the background current. Although the electrical signal attributed to the enzyme activity can be observed at both micro- and macro-electrodes, the spikes can be only identified at nano- and micro-electrodes, as the sensitivity of the current to the variation of the enzyme distance is related to the size of the detection electrode. At macroelectrodes, the spike is too broad to be identified and hence it is difficult to observe spikes from the background.

In addition, the characterisation of the spikes is also influenced by the bandwidth of the measurement electronics. Fig. 7a–f are calculated for different filter models, *i.e.* different potentiostats, which feature different transfer characteristics and different bandwidths. If the same series of impact events was recorded simultaneously with both potentiostats, different current responses would hence be observed and the average spike duration and the number of spikes detected may alter between the measurements. The filter response to signals in the high frequency regime is herein particularly interesting as series of signal fluctuations in this regime may be resolved through some potentiostats and then be identified as individual spikes, while a different potentiostat may show the same series of fluctuations as a single longer spike. The application of a low-pass filter operated at a cut-off frequency of 4 kHz therefore leads to a shorter average spike length and a larger number of spikes being detected if compared to a measurement using a low-pass filter set at 50 Hz.

## 4 Conclusions

It is computationally shown that in principle the nano-impact method enables the electrochemical characterisation of freely-diffusing enzymes if a small electrode is used, the potentiostats bandwidth is sufficient, and the enzyme features a large average turnover number. These findings apply to an enzyme operating at a constant turnover number, while fluctuations in the enzyme activity will further enhance its detectability. The model presented provides understanding of the enzyme-electrode system and useful predictions for experimentalists: we demonstrate that current responses corresponding to single catalase activity can in principle be observed at electrodes with radii varying from a few nanometres to a few micrometres. However, the simulated current spikes are too small to be distinguished from the background noise in any real experiment using a broad bandwidth. Enzymes with faster turnover numbers than catalase lead to larger current signals that can be experimentally observed and electrodes with smaller sizes better detect the signals. Again, the influence of the measurement electronics cannot be ignored. The electronics with a short bandwidth keeps more information than that of a broad bandwidth and is more favourable in the detection of the single enzyme activity.

The model is applied to simulate current signals that could possibly be attributed to single catalase at a 5.0 μm electrode measured at a cut-off frequency of 4 kHz. The simulation and the experiment show however significant discrepancy in the magnitude and the duration time of the current signal, revealing that without further consideration of the enzyme catalysis kinetics and the influence of the experiment environment, the experimental phenomena cannot be explained as the detection of product generated by the activity of a single catalase enzyme in solution.
