# Supplementary material for: Understanding single enzyme activity via the nano-impact technique
Source: Chem Sci. 2017 Jul 19;8(9):6423–32. doi: 10.1039/c7sc02084h (PMC5632796; doi:10.1039/c7sc02084h)
Supplement: Supplementary file 1 [file SC-008-C7SC02084H-s001.pdf]

Electronic Supplementary Material (ESI) for Chemical Science.
This journal is © The Royal Society of Chemistry 2017

Supporting Information of "Understanding Single Enzyme Activity via the Nano-Impact Technique"

Chuhong Lin, Enno Kätelhön, Lior Sepunaru, Richard G. Compton*

Department of Chemistry, Physical and Theoretical Chemistry Laboratory, Oxford University, South Parks Road, Oxford OX1 3QZ, UK

*To whom correspondence should be addressed

Email: richard.compton@chem.ox.ac.uk

Phone: +44 (0) 1865 275957 Fax: +44 (0) 1865 275410

S1 Simulation Mesh

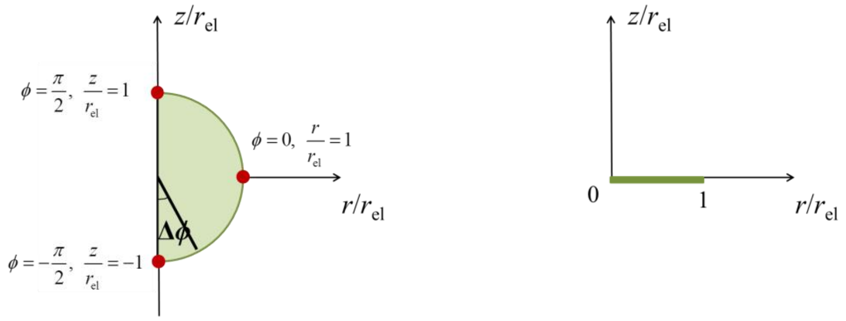

Figure S1 Illustration of the micro-sphere (a) and micro-disc (b) in two-dimensional cylindrical coordinates.

As the enzyme-electrode system is symmetric to the z-axis, only the simulation space $r \geq 0$ needs to be taken into consideration. Figure S1a shows the spherical electrode in the two-dimensional cylindrical coordinate system. In the finite difference method, the half circle representing the sphere electrode in the simulation space is evenly divided into n_ϕ parts. To make the simulation simple, n_ϕ is usually selected to be an even value. The angle ϕ of the half circle is distributed evenly over the range $\left[-\frac{\pi}{2}, \frac{\pi}{2}\right]$:

$$\phi_j = -\frac{\pi}{2} + (j-1)\Delta\phi,\ \Delta\phi = \frac{\pi}{n_\phi},\ j = 1, 2, \ldots, n_\phi, n_\phi + 1 \tag{1}$$

The mesh for the area $\{z/r_{\mathrm{el}} \in [-1,\ 1], r/r_{\mathrm{el}} \in [0,\ 1]\}$ is then determined by:

$$\frac{r_{\mathrm{i}}}{r_{\mathrm{el}}} = -\sin(\phi_{\mathrm{i}}),\ i = 1, 2, \ldots, \frac{n_\phi}{2}, \frac{n_\phi}{2} + 1 \tag{2}$$

$$\frac{z_{\mathrm{j}}}{r_{\mathrm{el}}} = \cos\left(\phi_j\right),\ j = 1, 2, ..., n_\phi, n_\phi + 1 \tag{3}$$

In r direction, for the region $r > r_{\mathrm{el}}$, the expanding grid is:

$$\frac{r_{\mathrm{i}}}{r_{\mathrm{el}}} - \frac{r_{\mathrm{i\text{-}1}}}{r_{\mathrm{el}}} = \Delta r \cdot w^{i-\left(\frac{n_\phi}{2}+1\right)-1}, \Delta r = \sin\left(\Delta\phi\right), i = \frac{n_\phi}{2} + 2, \frac{n_\phi}{2} + 3, ... \tag{4}$$

where w is the expandion factor selected to be 1.03 in the simulations of this work. The convergence test on w can be found in Section S2.

In z direction, a similar expanding grid is built for the region $z < -r_{\mathrm{el}}$:

$$\frac{z_{\mathrm{j}}}{r_{\mathrm{el}}} - \frac{z_{\mathrm{j+1}}}{r_{\mathrm{el}}} = -\Delta z \cdot w^{-j}, \Delta z = 1 - \cos\left(\Delta\phi\right),\ j = 0, -1, ... \tag{5}$$

The same expandion factor $w = 1.03$ is applied.

Due to the presence of the enzyme, the grids built in positive z direction are more complex. In the region $z \in \left(r_{\mathrm{el}}, r_{\mathrm{el}} + d_{\mathrm{enzyme}}\right]$, as the concentration gradient changes dramatically at the enzyme location, the grid is built as follows:

$$\frac{z_{\mathrm{j}}}{r_{\mathrm{el}}} - \frac{z_{\mathrm{j\text{-}1}}}{r_{\mathrm{el}}} = \begin{cases} \Delta z \cdot w^{j-\left(n_\phi+1\right)-1}, r_{\mathrm{el}} < z_{\mathrm{j}} \le r_{\mathrm{el}} + \dfrac{d_{\mathrm{enzyme}}}{2} \\ \Delta z \cdot w^{n_{\mathrm{e}}-\left(j-\left(n_\phi+1\right)\right)}, r_{\mathrm{el}} + \dfrac{d_{\mathrm{enzyme}}}{2} < z_{\mathrm{j}} \le r_{\mathrm{el}} + d_{\mathrm{enzyme}} \end{cases} \tag{6}$$

where n_{e} is the index of z_{j} corresponding to $z_{\mathrm{j}} = r_{\mathrm{el}} + d_{\mathrm{enzyme}}$.

For the rest of z direction $z > r_{\mathrm{el}} + d_{\mathrm{enzyme}}$, an expanding grid is applied:

$$\frac{z_{\mathrm{j}}}{r_{\mathrm{el}}} - \frac{z_{\mathrm{j\text{-}1}}}{r_{\mathrm{el}}} = \Delta z \cdot w^{j-n_{\mathrm{e}}-1}, j = n_{\mathrm{e}} + 1, n_{\mathrm{e}} + 2, ... \tag{7}$$

An illustration of the disc electrode in (r, z) coordinates is shown in Figure S1b. To keep consistency with the spherical model, the calculation grid of the enzyme-disc system in r direction is the same as that of the enzyme-sphere system, determined by Eq(2) and Eq(4). In the z direction, when $z < r_{\mathrm{el}}$, the grid is calculated as:

$$\frac{z_{\mathrm{j}}}{r_{\mathrm{el}}} - \frac{z_{\mathrm{j\text{-}1}}}{r_{\mathrm{el}}} = \begin{cases} \Delta z \cdot w^{j-2}, 0 < z_{\mathrm{j}} \le \dfrac{d_{\mathrm{enzyme}}}{2} \\ \Delta z \cdot w^{n_{\mathrm{e}}-(j-1)}, \dfrac{d_{\mathrm{enzyme}}}{2} < z_{\mathrm{j}} \le d_{\mathrm{enzyme}} \end{cases}, \ z_1 = 0 \qquad (8)$$

For the grids in the region $z > d_{\mathrm{enzyme}}$, Eq.(7) is applied.

S2 Convergence Test of the Simulation for the Enzyme Catalysis Model

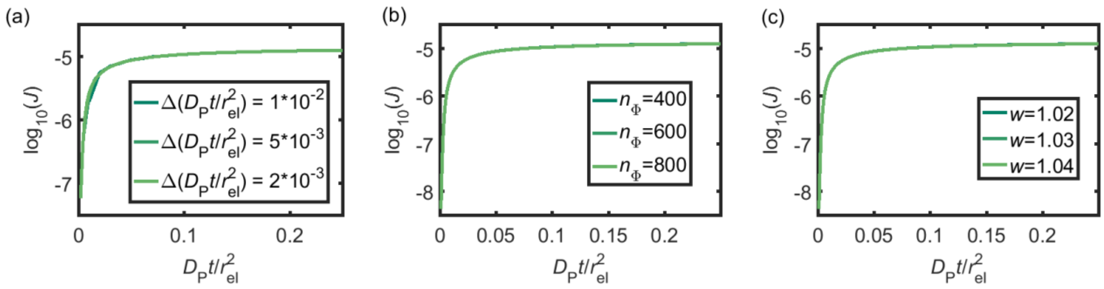

Figure S2 Convergence check of (a) the simulation time interval, (b) the number of grid points on the half sphere, (c) the expanding factor for the enzyme-microdisc model. D_{P} is 10^{-9} m^2 s^{-1} and r_{el} is 1 μm.

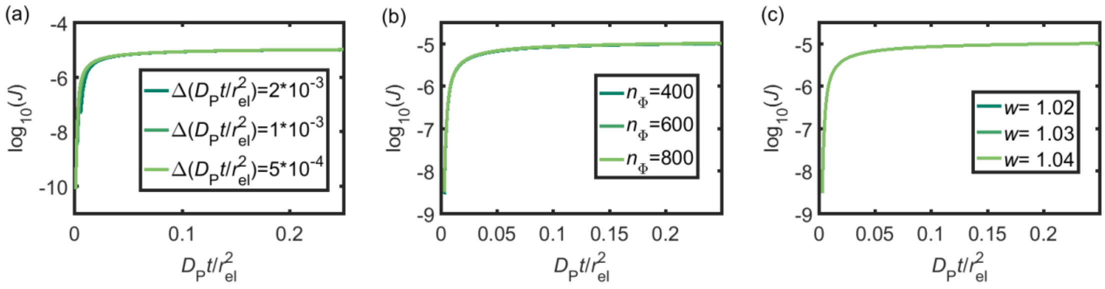

Figure S3 Convergent check of (a) the simulation time interval, (b) the number of grid points on the half sphere, (c) the expanding factor for the enzyme-microsphere model. D_P is 10^{-9} m^2 s^{-1} and r_{el} is 1 μm.

In Figure S2 and Figure S3, the reaction flux (J) caused by the redox reaction of the enzyme product P at the microdisc (S2) and the microsphere (S3) electrode are calculated with a series of simulation time intervals $\Delta(D_P t/r_{el}^2)$ and simulation grids. The simulation girds are determined by two parameters: the number of grid points on the half sphere surface [-π/2, π/2] n_ϕ and the expanding factor w. It is proved that for both the enzyme-microdisc and the enzyme-microsphere models, the simulation results are independent on the value of n_ϕ when these simulation parameters are small enough to reach convergent states.

S3 Enzyme-Microsphere Geometry

Figure S4 shows the characterisation of the enzyme-microsphere system. Similar to Figure 3 in the main text, similar responses to the variation of the catalytic ability K_{cat} and the relative distance from the electrode d can be observed. Figure S5 is the working curve of the normalized steady-state current for the enzyme-microsphere system. Compared with the working curve collected from the microdisc electrode, the collecting efficiency is slightly smaller at a microsphere electrode with identical radius. This is because, when the enzyme is located on the z-axis, the disc geometry allows the electrode interacting with more product

species than the sphere geometry. But as the difference is not significant between the enzyme-microdisc model and the enzyme-microsphere model, the results obtained from the two microelectrodes are approximately the same. Therefore in the main text, only the simulation for the enzyme-microdisc electrode is presented.

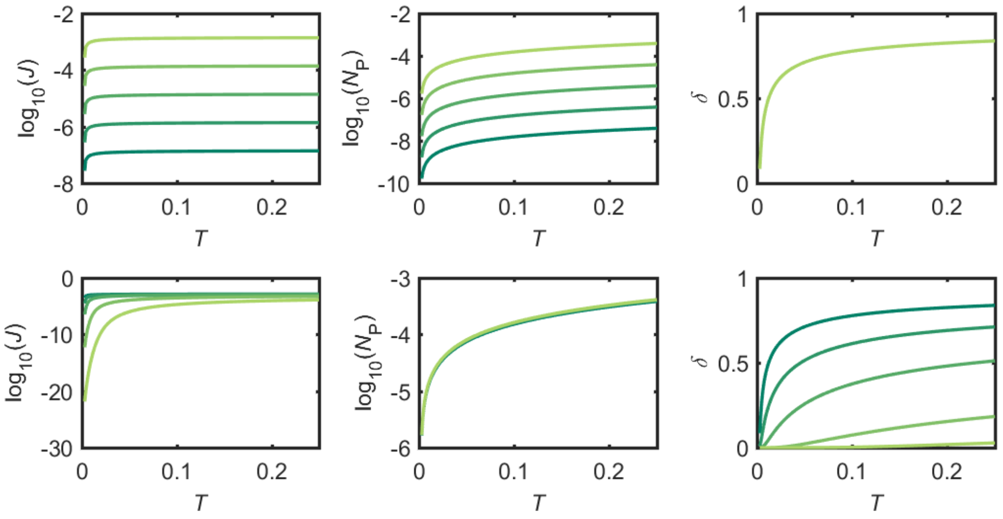

Figure S4 Characterisation of the enzyme-microsphere system. (a), (b) and (c) are the current flux *J*, the total amount of product N_P, and the collecting efficiency *σ* varying with the reaction time *T* under different catalytic ability K_{cat}. K_{cat} varies from 10 to 10^5. *d* = 0.05. (d), (e) and (f) are the current flux *J*, the total amount of product N_P, and the collecting efficiency *σ* varying with the reaction time *T* under different enzyme location *d*. *d* varies from 0.05 to 1.0. $K_{cat} = 10^5$.

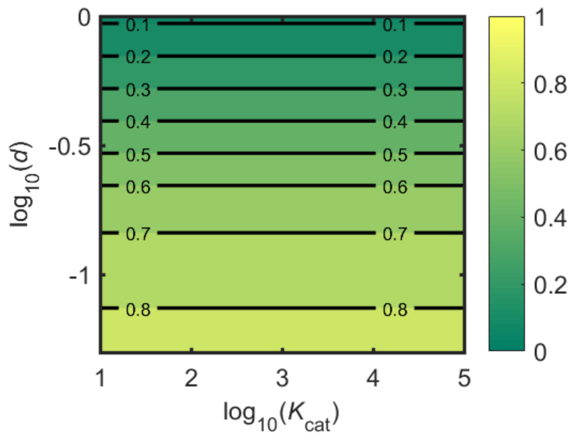

Figure S5 Normalized steady-state current (defined as the current at *T* = 0.25) as a function of *d* and K_{cat} for the enzyme-microsphere system.

S4 Convergence Test of the Electrode Response of a Freely-Diffusing Enzyme

Via the combined finite difference simulation of a stationary enzyme and the movement of a freely-diffusing enzyme as described by the hindered diffusion theory, the current signal caused by the solution-phase catalytic reaction of an enzyme can be simulated. The simulation of this whole enzyme-electrode system is tested under different simulation parameters: the random walk step length Δt_{rw}, during which the enzyme is regarded stationary and the catalytic reaction occurs at a certain location; the maximum simulation time for one enzyme location t_{max}; and the simulation space $d_{enzyme} = [d_{min}, d_{max}]$. In addition, to simulate the experimental situation, a low-pass filter with a cutoff frequency f_{cutoff} is applied, which influences the convergence of a simulation. Here the average half-spike width is selected to characterize the random current signals collected in the chronoamperogram. An example is shown in Figures S6 and S7 depicting the convergence test for the 0.5 μm electrode in Figure 7. Figure S6 compares the simulation results at different t_{max} and Δt_{rw}. It shows that when the random walk step length is short and the maximum simulation time for one position is long, similar stochastic spikes are obtained. Figure S7 studies the influence of

the simulation space. The expansion of the simulation space does not make much difference on the spike duration for the 0.5 μm electrode.

To simulate the experimental data, the results are required to be independent of t_{max} and Δt_{rw}, which we have examined for all simulation results in Figures 7 and 8. However, the influence of the simulation space remains an input parameter, as the closest approach d_{min} is limited under real experimental conditions. For instance, as the enzyme undergoes a solution-phase catalytic reaction, the closest approach should be larger than the size of the enzyme but the exact distance cannot be determined. The value of the upper boundary d_{max} determines the frequency of observed spikes. Considering the simulation time it may cost, the upper boundary cannot be selected as far as possible. In the comparison among the simulation results at different microelectrodes as shown in Figure 7, the simulation space is therefore defined identically from 5 nm to 5 μm for all electrode sizes. It can be found in Figure S7 that the simulation space is large enough to describe the movement of the enzyme around a 0.5 μm electrode. But as the collecting efficiency is determined by the relative distance d_{enzyme}/r_{el} rather than d_{enzyme}, the closest approach 5 nm may be too large for smaller electrodes to be convergent and the upper boundary 5 μm may be too small for larger electrodes. When theoretically investigating the difference between the electrodes of various sizes, it is however necessary to keep the simulation space consistent for all the enzyme-electrode systems as done in Figure 7. When simulating real experimental conditions, the simulation space tough needs to be carefully selected to make the results converge as it was done in Figure 8, where a greater value was modelled.

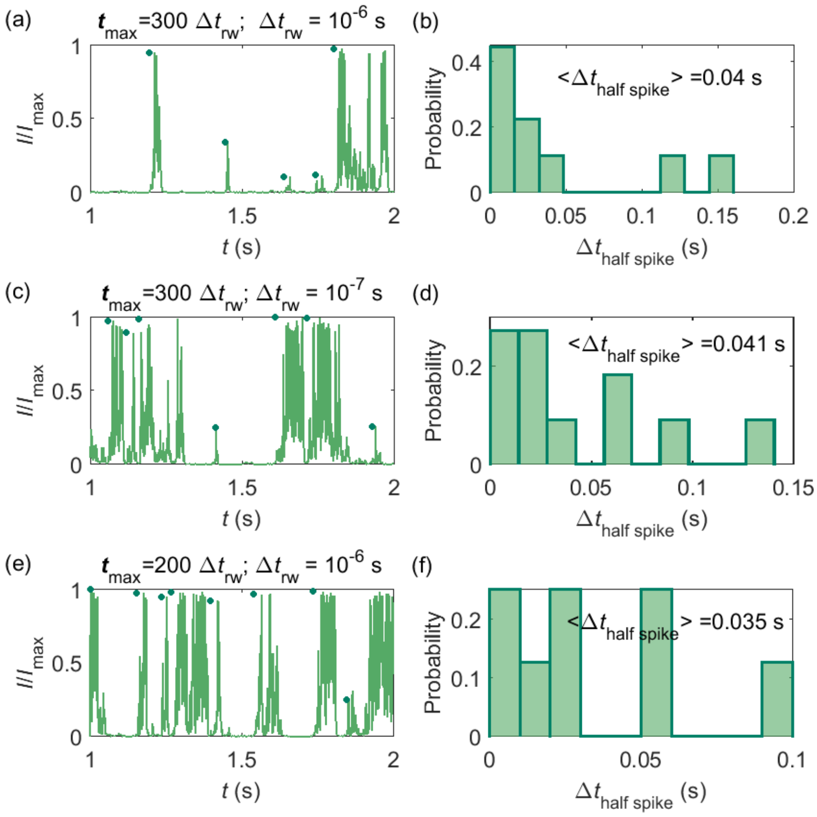

Figure S6 Convergence test of t_{max} and Δt_{rw} for the 0.5 μm electrode in Figure 7.

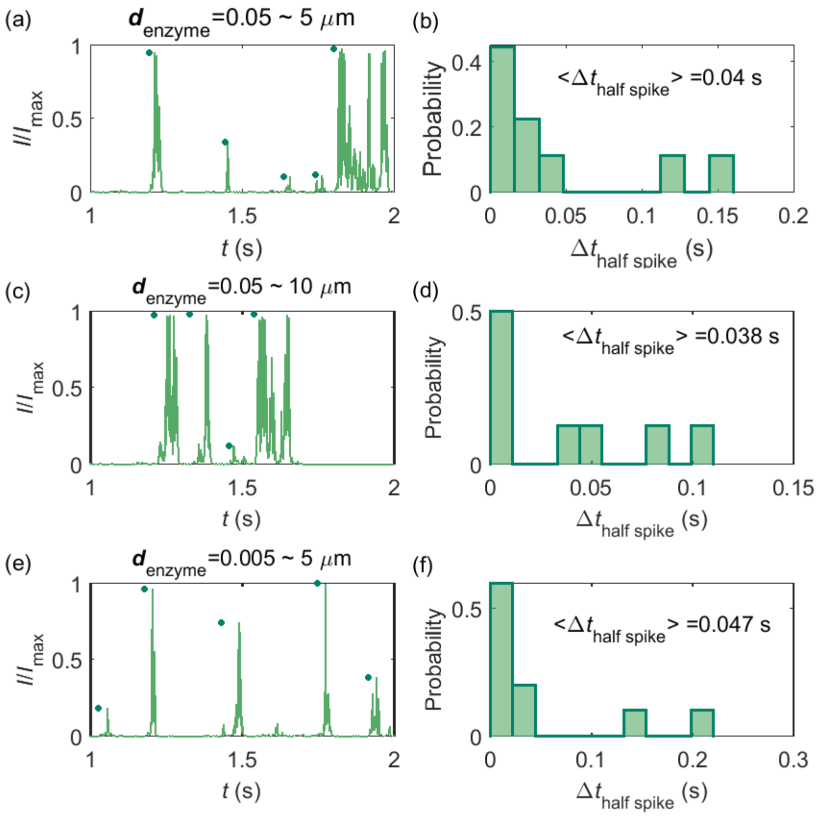

Figure S7 Convergence test of the simulation space for the 0.5 μm electrode in Figure 7.

S5 Control Experiment to Figure 8a

The control experiment to Figure 8a, in the absence of catalase, is shown in Figure S8. The other experimental conditions are the same as applied in Figure 8a.

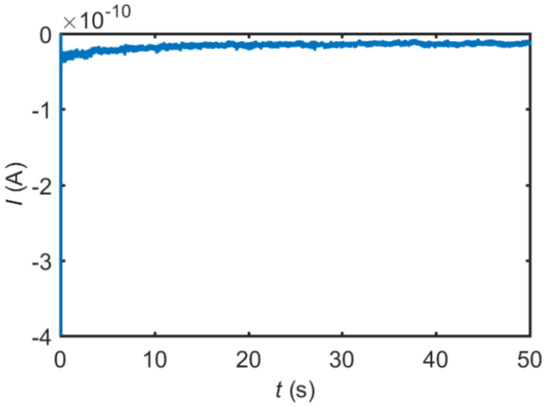

Figure S8 Control experiment relating to Figure 8a.
